# The Effects of Information Continuity and Interpersonal Continuity on Physician Services Online: Cross-sectional Study

**DOI:** 10.2196/35830

**Published:** 2022-07-21

**Authors:** Yan Xuan, Chaojin Guo, Wei Lu

**Affiliations:** 1 Department of Nursing Hainan Women and Children’s Medical Center Hainan China; 2 School of Management Hainan Medical University Haikou China

**Keywords:** continuity of care, web-based medical service, service quality, information continuity, interpersonal continuity

## Abstract

**Background:**

Web-based medical services have become an effective supplement to traditional services in hospitals and an essential part of medical services. Studies have shown that web-based medical services are useful for shortening the delayed admission time and for enhancing the treatment effect from the service continuity perspective. However, the specific measures that patients and physicians should take to improve service continuity remain unknown.

**Objective:**

Based on the information richness theory and continuity of care, this study investigates the dynamic effects of information continuity and interpersonal continuity on physician services online.

**Methods:**

Data of 7200 patients with 360 physicians covering complete interaction records were collected from a professional web-based platform in China. Content analysis was performed to recognize matching patients and physicians, and least square regression analysis was performed to obtain all empirical results.

**Results:**

Empirical results showed that in the short term, information continuity (including offline experience, medical records, and detailed information) influences physicians’ web-based services, and their influences show heterogeneity. Moreover, if a patient’s online physician is the same physician who he/she has visited offline, we find that interpersonal continuity is important for service. In the long term, information continuity and interpersonal continuity positively improve service continuity by facilitating repeat purchases.

**Conclusions:**

Overall, our findings not only shed new light on patient behavior online and cross-channel behavior but also provide practical insights into improving the continuity of care in online health communities.

## Introduction

### Background

High continuity of care is the key to improving medical service quality and decreasing irrational use [1], which is an important theme of digital transformation that is receiving increasing attention. Currently, there is no universal definition of the concept and characteristics of continuity of care. However, experienced continuity, information continuity, coherence of medical records, cross-boundary and team continuity, longitudinal continuity, and interpersonal continuity are widely recognized as important elements of continuity of care [2]. As some medical services can be done using information technology, such as appointments and treatments, the use of information technology in health care could realize the mutual recognition of inspection results and sharing of medical records, thus improving the continuity of care [3].

Online health communities provide a channel for patient and physician contact conveniently by overcoming space-time limits and enriching information provision [4,5]. Web-based medical services have become an effective supplement to traditional services in hospitals and an essential part of medical services [6]. Patients can communicate with physicians via various types of services, including individual service (written consultation, phone consultation, video consultation) and team service. No matter which service patients use, they should post their questions mandatorily and provide offline treatment materials selectively if they have them. This offline information helps improve continuity of care and is useful for physicians to make an accurate diagnosis.

From the continuity of care perspective, “internet plus medical service” (a new application of the medical industry, which includes internet as the carrier and the technical method of health education, medical information query, electronic health records, disease risk evaluation, online consulting, electronic prescription, remote consultation, remote treatment and rehabilitation, and other forms of health care services) is believed to integrate medical treatment, health care, and rehabilitation, with extending medical services outside the hospital. The form of “internet plus medical service” is changing from “split” to “holistic medical treatment,” and this treatment plays a significant role in interpersonal continuity, information continuity, and geographical continuity [7]. However, the above benefits are only theoretical judgments and there are no empirical studies to examine the role of online health communities. To the best of our knowledge, this study is among the first to investigate the effects of information providing from the continuity of care perspective. Although the literature on online health communities is abundant [8-11], they rarely focus on the influence of offline experiences on online behaviors. In addition, prior studies have revealed that web-based medical services are useful for shortening the delayed admission time and for enhancing the treatment effect from the service continuity perspective [1]. However, the specific measures that patients and physicians should take to improve service continuity remain unknown. Based on the information richness theory and continuity of care, this study aims to investigate the dynamic effects of information continuity and interpersonal continuity on physicians’ services online. To fill the above research gap, we follow patient’s online information-providing behavior to examine the following research questions.

Research question 1: How does information continuity (offline treatment experience, medical records, and detailed information provision) influence physicians’ web-based services?

Research question 2: How does interpersonal continuity influence physicians’ web-based services?

### Theoretical Foundation and Hypothesis Development

#### Information Richness Theory

Information richness theory, also called as media richness theory, takes the communication channel as an objective feature to determine the ability of information transmission [12]. It describes the ability to change people’s understanding within a time interval and consists of 4 standard features: the ability to give immediate feedback, the ability to communicate multiple social cues, linguistic diversity, and personalization [13]. The amount of information affects the communication outcomes by reducing uncertainty [14]. The appropriate amount is determined by the purpose of the communication and the content. The rich information can provide practical help for communication, coordination, collaboration, and information sharing. With the development of media, the standards for evaluating information richness have changed and a large number of important research results have been gained. Users’ perceived information richness would affect their satisfaction [15] and continued willingness to use [16]. Moreover, interactivity is an important factor in assessing the perceived richness of information [15,17,18] and could determine the platform development [19]. High interactivity would increase the willingness of users to use media or services [20,21]. High richness could decrease consumers’ uncertainty in online retail and increase their loyalty [22]. In the health field, the essential difference between web-based medical services and traditional medical services (ie, face-to-face) is information richness. However, with the development of web-based services, studies find that web-based psychological interventions are as effective as face-to-face psychotherapy [23]. For sensitive diseases, patients prefer a high information richness channel such as face-to-face therapy [24]. High information richness improves users’ perception of knowledge quality, source credibility, and knowledge consensus, especially under high health threats [25].

#### Continuity of Care

Service continuity was first proposed in the Folsom Report, Millis Report, and Willard Report in 1966, and then its concept has been developed and enriched. Subsequently, scholars have elaborated on various dimensions of continuity of care [2,26]. Continuity of care has also been defined in related studies as repeated contact between patients and physicians [27]. For the service provider, continuity of care can be divided into information continuity, multi-department continuity, time continuity, interpersonal continuity, and management continuity [2]. For the service receiver, experience continuity and geography continuity are important dimensions of continuity of care. The most widely used dimensions are information continuity, time continuity, and interpersonal continuity [28,29].

Information continuity means that different medical institutions have complete, timely, shareable, mutually recognized, and cohesive information in the aspects of disease prevention, examination, diagnosis, treatment, and rehabilitation of patients [2]. The health care provider uses information on past events to deliver care that is appropriate to the patient’s current circumstance [26]. Interpersonal continuity means providers develop an ongoing relationship with patients and the provider has knowledge of the patient as a person [26]. Interpersonal continuity is built on repeated (but not necessarily exclusive) contacts and is important for building trust and respect. The central skill fostered by interpersonal continuity over time is the ability to make and value a multidimensional diagnosis based on the biopsychosocial model within the patient’s context [2,30]. As many patients nowadays have more than one preferred health care provider, when transitions in care occur, communication and collaboration between health care providers (ie, information continuity) are more important than interpersonal continuity [31]. Continuity of care is associated with patient satisfaction, adherence to medical advice, and the use of hospital services [1]. Medical care is a special service for maintaining health; the continuity of life determines that medical care must be continuous. In the context of population aging, disease spectrum change, rapidly rising medical costs, and patients’ increasing emphasis on self-worth, continuous medical care has become the focus of the establishment and improvement of health service systems in various countries.

#### Information Provision and Medical Service

The specialty of medical service leads to high information asymmetry between physicians and patients. It is difficult for both physicians and patients to fully explain the health condition within a limited time. Medical service is directly related to the health or safety of patients; thus, they often visit several physicians for rich information. Rich information helps improve physician-patient interaction and patient experience, thereby enhancing the information service capability and user satisfaction [32]. Quantitative information on the quality of health services can be more useful to patients by combining digital information with graphics [33]. Physicians’ information has an important impact on the patient’s decision [34].

Since 1998, the government and private sectors have recognized the importance of using technology for improving care delivery and have made progress in setting the stage for transforming health care delivery through vastly improved use of health information technology [35]. There have been many government eHealth initiatives aiming to improve continuity and coordination through information, such as Personally Controlled Electronic Health Record [36], electronic health records [37], and telemedicine [38]. Although the use of online health communities is thought to help improve the continuity of care [3], only few empirical studies have been conducted to explore these influence mechanisms.

Online health communities serve as a bridge to help patients and physicians solve the problem of information asymmetry and improve the physician-patient relationship [39]. There are mainly 2 types of patients in online health communities. One type is those who have not seen a physician in hospitals and hope to receive advice on care through the web-based platform. The other category is the patients who have already seen a physician in hospitals and hope to receive more advice for disease treatment, rehabilitation, prognosis, and interpretation of the test report after receiving diagnosis and treatment offline. For the second type, as patients have received medical service in the hospital, they have more information, which they can provide to physicians in online health communities to improve continuity of care. Higher continuity is associated with higher quality of health care [40]. Based on the dimensions of continuity of care, we propose the following hypotheses:

1. Hypothesis 1: High information continuity helps improve a physician’s web-based service. Previous studies have indicated that trust could change in different periods dynamically. In the case of medical service, the roles of information continuity and interpersonal continuity may change as the physician contacts patients over time [41]. Therefore, we examined the effects of information continuity in the short term (for the current purchase) and in the long term (for the future purchases). In short term, response speed, information quality, and interaction quality have been widely used in prior studies [8,9]. Repeat purchase is often used to measure the long-term effects [42]. Therefore, we included them and developed the following hypotheses. Hypothesis 1a: (short-term) high information continuity would improve the response speed of a physician’s reply. Hypothesis 1b: (short-term) high information continuity would improve the information quality of a physician’s reply. Hypothesis 1c: (short-term) high information continuity would improve the interaction quality of a physician’s reply. Hypothesis 1d: (long-term) high information continuity would increase a patient’s repeat purchase. Patients with a close continuous relationship with a specific physician are more likely to receive the recommended care [43]. Service content and service quality of health care can vary substantially across channels. Therefore, patients engaging in multiple visits with the same physician could help obtain a continuous and satisfactory outcome [44]. Based on the above arguments, we hypothesize that if it is the same physician online and offline, the effects of information continuity on the physician’s service would be enhanced.
2. Hypothesis 2: High interpersonal continuity would enhance the relationships between information continuity and a physician’s service. Based on the richness of information, we recognize whether a patient has offline treatment experience and has told the online physician, and then, we recognize whether a patient has provided his offline medical records to the online physician, and we calculate the degree of information provision.

**Figure 1:**
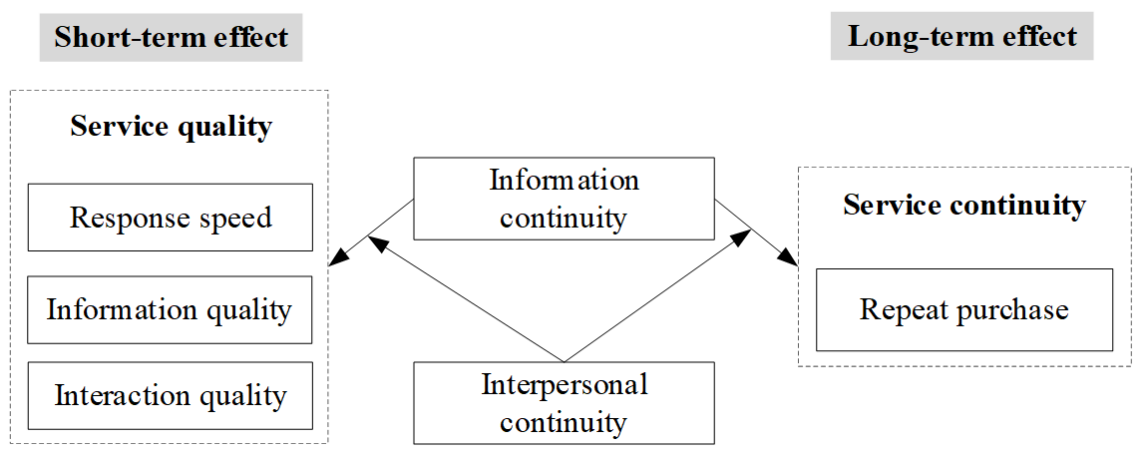
Conceptual model.

The conceptual model for the abovementioned hypotheses is shown in Figure 1.

## Methods

### Ethics Approval

This study was approved by the institutional review board of Hainan Women and Children’s Medical Center (HNWCMC202262).

### Research Context and Data Collection

**Figure 2:**
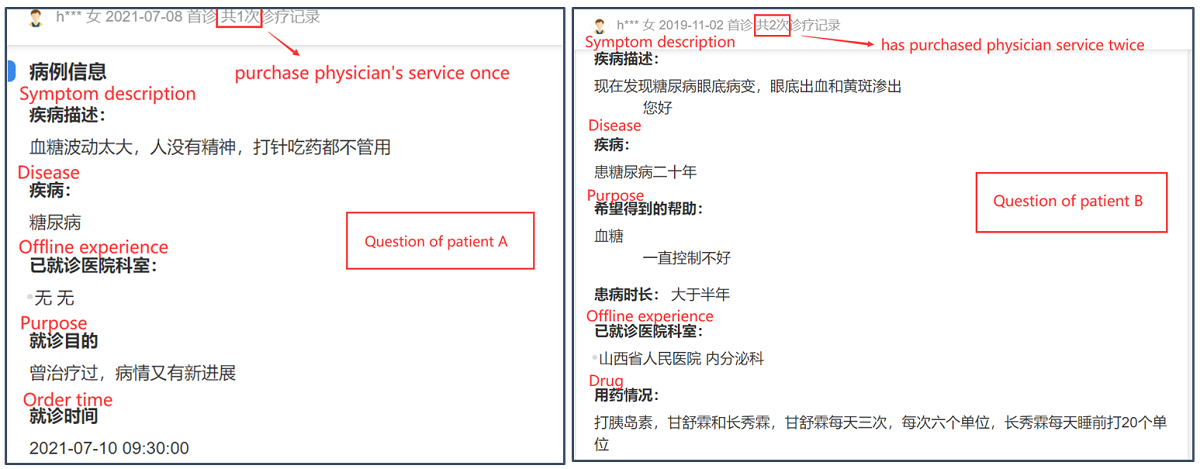
Examples of patients' questions.

**Figure 3:**
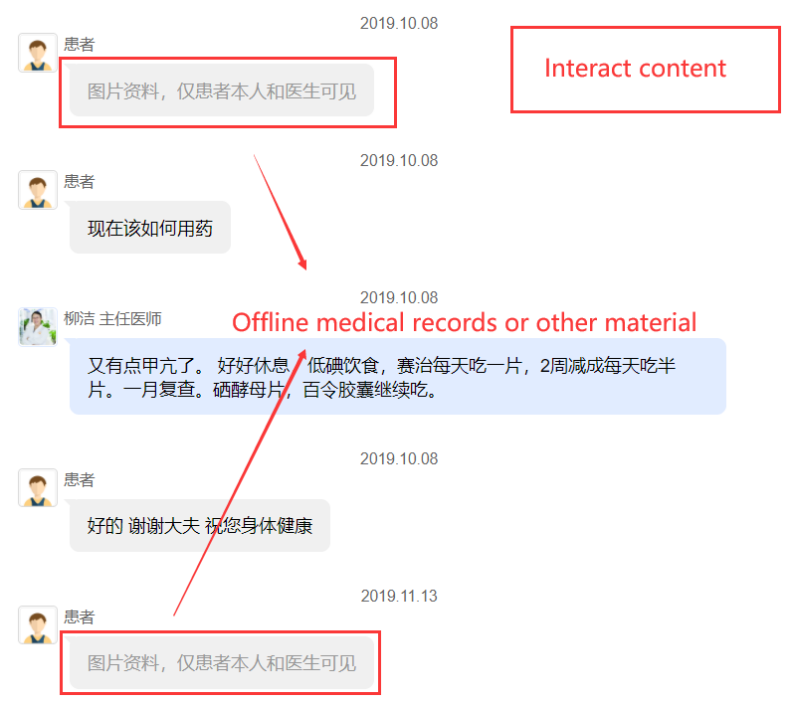
An example of interaction content.

We collect data from one of the most professional and popular online health communities in China: Haodf.com [45]. Haodf.com was founded in 2006 and is one of China’s leading online health care platforms. Haodf.com provides services such as hospital/physician information query, written consultation, phone consultation, video consultation, outpatient appointment, postdiagnosis disease management, family physician, disease knowledge, and popularization, and is widely trusted by physicians and patients. Haodf.com has a large number of high-quality physicians. By July 2021, Haodf.com had collected the information of more than 790,000 physicians in nearly 10,000 regular hospitals across the country. Among them, more than 240,000 physicians had registered on the platform, and those from AAA hospitals accounted for 73% of these active physicians. The hospitals in China are divided into 10 levels, and AAA is the best level. As of July 2021, Haodf.com has served more than 72 million patients. This online health community provides a physician-patient interaction platform for various diseases. Both individual services (eg, written consultation, phone consultation, video consultation) and team services are provided. Based on the aims of this study, we chose written consultation service and focused on physician-patient interaction content on diabetes for the following 2 reasons. First, chronic diseases have a long treatment period, and the patient often needs repeated communication with physicians. On Haodf.com, there is a large diabetic population, which was beneficial for the conduct of this research. Second, different from phone and video consultations, all interaction contents between physicians and patients based on written consultation are recorded on Haodf.com and shown publicly. We can obtain all the information that a patient has provided to his physician. By developing a web crawler, we firstly collected physician data from physician lists on Haodf.com, and 360 physicians were included. Then, for each physician, 20 complete physician-patient interaction contents were collected, including symptom description, offline experience, purchase times, medical records, or other material provision (shown in Figures 2 and 3). Finally, data of 7200 patients with 360 physicians were included in the empirical study.

### Variables and Models

#### Dependent Variables

Four dependent variables were used to measure physicians’ services: response speed (*RS_ij_*), information quality (*InfQ_ij_*), interaction quality (*IntQ_ij_*), and repeat purchase (*RP_ij_*). Response speed, information quality, and interaction quality, which were often used to measure the quality of physicians’ online services in prior studies [34], were used to measure the short-term influence. The repeat purchase was used to measure the long-term influence.

#### Independent Variables

Information, including medical history, laboratory results, radiographs, and current diagnoses, as well as the history of medications and treatments, should be available to clinicians at the point of care whenever and wherever they need them, no matter where they were originally obtained [35]. Therefore, considering the information provision in online health communities, 3 independent variables were included to measure patient *i*’s offline information provision. Based on the degree of offline information provision, we measured whether patient *i* had offline experience (*OE_ij_*) and mentioned it during the online consultation with physician *j*. If that was so, we measured the number of offline medical records or other material (*OMR_ij_*) that patient *i* had provided to support the online service of physician *j*, and the number of words (*ODI_ij_*) that patient *i* has described his offline experience to online physician *j*. These 3 variables describe the information continuity.

#### Moderating Variable: Interpersonal Continuity

Based on the interaction content, we recognized whether the physician in the patient’s offline experience is the same as the physician who patient *i* had consulted online (*SP_ij_*), and used a dummy variable in empirical models. This variable describes interpersonal continuity.

#### Control Variables

Other important information about physicians that may influence physician service was also included to control: physician medical title (*MTitle1_j_* and *MTitle2_j_*), physician education title (*ETitle_j_*), physician online reputation (*POR_j_*), and hospital level (*Level_j_*). More details can be found in Table 1. Table 1 shows the definitions of the variables in the empirical analysis and their measurements. The unit of analysis is the individual online health community patient-physician interaction.

Accordingly, our empirical models are shown in 
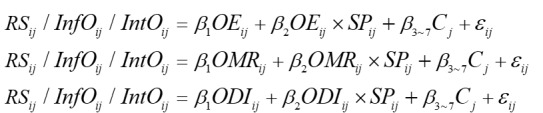
, showing short-term effects and 
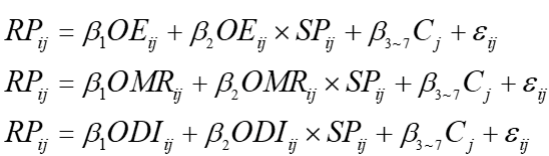
, showing the long-term effects, where *i*=1,…, *N* represents the patients, *j*=1,…, *M* represents the physicians, *β*_1_ to *β*_7_ are the focus parameters to be estimated. C represents control variables. *ε* is the error term associated with observation *i* and *j*.

**Table 1:** Description of the variables.

Variables			Description		Measures
**Dependent variables**					
	Response speed (RS_ij_)	The response time that physician *j* could reply to a patient’s question in 24 hours.		Use response time directly. The value is in days.	
	Information quality (InfQ_ij_)	The level of detail in the physician *j*’s reply for patient *i*.		The number of words replied.	
	Interaction quality (IntQ_ij_)	The frequency of physician-patient interaction.		The number of interactions between patient *i* and physician *j* is used.	
	Repeat purchase (RP_ij_)	Patient *i* may have purchased physician *j*’s service many times.		A dummy variable that describes whether patient *i* has repurchased physician *j*’s service.	
**Independent variables**					
	Offline experience (OE_ij_)	Patient *i* may have gone to a hospital for treatment before consulting online.		A dummy variable that describes whether patient *i* has provided his offline experience to the online physician. “1” refers to yes, and “0” refers to no.	
	Offline medical records (OMR_ij_)	Patient *i* may have gone to a hospital for treatment before consulting online, and undergo some tests.		The number of results of tests that patient *i* has provided to the online physician.	
	Offline detailed information (ODI_ij_)	Patient *i* may have gone to a hospital for treatment before consulting online.		The number of words that patient *i* has described his offline experience to the online physician.	
**Moderating variable**					
	Same physician (SP_ij_)	Whether the physician in patient’s offline experience is same as the physician who patient *i* has consulted online.		A dummy variable that describes whether it is the same physician online and offline. “1” refers to yes, and “0” refers to no.	
**Control variables**					
	Physician medical titles (MTitle1_j_ and MTitle2_j_)	Physicians have medical titles, which are evaluated by the medical government based on their medical skills in China, including chief physician, associate chief physician, attending physician, and resident physician.		A dummy variable that describes whether physician *j* is a chief physician or associate chief physician. “1” refers to physician *j* as a chief physician or associate chief physician, and “0” refers to other medical titles.	
	Physician education title (ETitle_j_)	Whether the physician *j* has worked at a university.		A dummy variable that describes whether physician *j* is a professor or associate professor at a university. “1” refers to physician *j* as a professor or associate professor, and “0” refers to other educational titles.	
	Physician online reputation (POR_j_)	The reputation is based on physician *j*’s online work.		An indicator (ranges from 0 to 5) that is calculated by the website based on patients’ feedbacks is used directly.	
	Hospital level (Level_j_)	Hospitals have levels that are evaluated by the medical government based on their comprehensive health care quality in China.		A dummy variable indicating if the hospital where physician *j* works is AAA hospital. “1” refers to physician *j* works in an AAA-level hospital, and “0” refers to other level hospitals.	

## Results

### Descriptive Statistics

Table S1 of Multimedia Appendix 1 shows the descriptive statistics and the correlations of the variables. On average, 46% (3312/7200) of the patients mentioned their offline experience. Each patient provided 6.11 offline medical records or other material and 38.5 words about the offline experience; 77% (5544/7200) of the patients chose the same physician online and offline. The response rate in 24 hours was 67.3% (242/360). The average numbers of information words and interactions were 12.61 and 17.97, respectively; 29% (2088/7200) of the patients purchased the physician service repeatedly. Multicollinearity is not an issue in our research as all variance inflation factors were less than 10.

### Empirical Results: Short-term Effects

The ordinary least squares was used to obtain our short-term effect results, which are shown in Table 2, Table 3, and Table 4.

**Table 2:** Results for information continuity (offline experience provision): short-term effects.

Variables	Response speed				Information quality				Interaction quality			
	Model 1^a^		Model 2^b^		Model 1^c^		Model 2^d^		Model 1^e^		Model 2^f^	
	*β* (SD)	*P* value	*β* (SD)	*P* value	*β* (SD)	*P* value	*β* (SD)	*P* value	*β* (SD)	*P* value	*β* (SD)	*P* value
Level	–.017 (.006)	.008	–.017 (.006)	.007	–.036 (.050)	.48	–.052 (.049)	.29	.022 (.003)	<.001	.022 (.003)	<.001
MTitle1^g^	–.021 (.008)	.006	–.019 (.008)	.01	.063 (.059)	.28	.152 (.058)	.009	.011 (.004)	.004	.010 (.004)	.009
MTitle2	–.002 (.007)	.80	–.002 (.007)	.83	.171 (.058)	.003	.188 (.057)	.001	.009 (.004)	.02	.009 (.004)	.02
ETitle^h^	–.017 (.007)	.01	–.017 (.007)	.01	–.120 (.052)	.02	–.117 (.051)	.02	.002 (.003)	.53	.002 (.003)	.53
POR^i^	.092 (.011)	<.001	.092 (.011)	<.001	1.930 (.082)	<.001	1.950 (.081)	<.001	–.105 (.005)	<.001	–.105 (.005)	<.001
OE^j^	N/A^k^	N/A	–.009 (.005)	.053	N/A	N/A	–.518 (.036)	<.001	N/A	N/A	.006 (.002)	.02

^a^Adjusted *R*^2^=0.010; *F*_5,7720_=16.808; *P*<.001.

^b^Adjusted *R*^2^=0.010; *F*_1,7719_=3.730; *P*=.053.

^c^Adjusted *R*^2^=0.090; *F*_5,7720_=153.226; *P*<.001.

^d^Adjusted *R*^2^=0.113; *F*_1,7719_=202.729; *P*<.001.

^e^Adjusted *R*^2^=0.052; *F*_5,7720_=85.391; *P*<.001.

^f^Adjusted *R*^2^=0.052; *F*_1,7719_=5.915; *P*=.02.

^g^MTitle1: physician medical title.

^h^ETitle: physician education title.

^i^POR: physician online reputation.

^j^OE: offline experience.

^k^N/A: not applicable.

**Table 3:** Results for information continuity (offline medical record provision): short-term effects.

Variables	Response speed				Information quality				Interaction quality			
	Model 1^a^		Model 2^b^		Model 1^c^		Model 2^d^		Model 1^e^		Model 2^f^	
	*β* (SD)	*P* value	*β* (SD)	*P* value	*β* (SD)	*P* value	*β* (SD)	*P* value	*β* (SD)	*P* value	*β* (SD)	*P* value
Level	–.017 (.006)	.008	–.013 (.006)	.04	–.036 (.050)	.48	.052 (.049)	.28	.064 (.015)	<.001	.097 (.014)	<.001
MTitle1^g^	–.021 (.008)	.006	–.017 (.008)	.03	.063 (.059)	.28	.151 (.057)	.008	–.135 (.017)	<.001	–.102 (.016)	<.001
MTitle2	–.002 (.007)	.80	–.001 (.007)	.86	.171 (.058)	.003	.186 (.056)	.001	–.016 (.017)	.35	–.010 (.016)	.52
ETitle^h^	–.017 (.007)	.01	–.014 (.007)	.04	–.120 (.052)	.02	–.054 (.050)	.29	.003 (.015)	.86	.028 (.014)	.05
POR^i^	.092 (.011)	<.001	.057 (.011)	<.001	1.930 (.082)	<.001	1.148 (.087)	<.001	–.208 (.024)	<.001	–.501 (.025)	<.001
OMR^j^	N/A^k^	N/A	–.001 (.000)	<.001	N/A	N/A	–.025 (.001)	<.001	N/A	N/A	–.009 (.000)	<.001

^a^Adjusted *R*^2^=0.010; *F*_5,7720_=16.808; *P*<.001.

^b^Adjusted *R*^2^=0.017; *F*_1,7719_=56.802; *P*<.001.

^c^Adjusted *R*^2^=0.090; *F*_5,7720_=153.226; *P*<.001.

^d^Adjusted *R*^2^=0.146; *F*_1,7719_=511.611; *P*<.001.

^e^Adjusted *R*^2^=0.037; *F*_5,7720_=59.550; *P*<.001.

^f^Adjusted *R*^2^=0.135; *F*_1,7719_=882.911; *P*<.001.

^g^MTitle1: physician medical title.

^h^ETitle: physician education title.

^i^POR: physician online reputation.

^j^OMR: offline medical record.

^k^N/A: not applicable.

**Table 4:** Results for information continuity (offline detailed information provision): short-term effects.

Variables	Response speed				Information quality				Interaction quality			
	Model 1^a^		Model 2^b^		Model 1^c^		Model 2^d^		Model 1^e^		Model 2^f^	
	*β* (SD)	*P* value	*β* (SD)	*P* value	*β* (SD)	*P* value	*β* (SD)	*P* value	*β* (SD)	*P* value	*β* (SD)	*P* value
Level	–.017 (.006)	.008	–.015 (.006)	.02	–.036 (.050)	.48	.032 (.009)	<.001	.064 (.015)	<.001	.068 (.014)	<.001
MTitle1^g^	–.021 (.008)	.006	–.022 (.007)	.003	.063 (.059)	.28	.023 (.011)	.03	–.135 (.017)	<.001	–.137 (.017)	<.001
MTitle2	–.002 (.007)	.80	–.006 (.007)	.40	.171 (.058)	.003	.003 (.011)	.74	–.016 (.017)	.35	–.025 (.017)	.14
ETitle^h^	–.017 (.007)	.01	–.013 (.007)	.05	–.120 (.052)	.02	.036 (.009)	<.001	.003 (.015)	.86	.011 (.015)	.46
POR^i^	.092 (.011)	<.001	.039 (.011)	<.001	1.930 (.082)	<.001	–.149 (.016)	<.001	–.208 (.024)	<.001	–.319 (.024)	<.001
OMR^j^	N/A^k^	N/A	.020 (.001)	<.001	N/A	N/A	.797 (.002)	<.001	N/A	N/A	.042 (.003)	<.001

^a^Adjusted *R*^2^=0.010; *F*_5,7720_=16.808; *P*<.001.

^b^Adjusted *R*^2^=0.047; *F*_1,7719_=297.243; *P*<.001.

^c^Adjusted *R*^2^=0.090; *F*_5,7720_=153.226; *P*<.001.

^d^Adjusted *R*^2^=0.370; *F*_1,7719_=4896.067; *P*<.001.

^e^Adjusted *R*^2^=0.037; *F*_5,7720_=59.550; *P*<.001.

^f^Adjusted *R*^2^=0.067; *F*_1,7719_=256.155; *P*<.001.

^g^MTitle1: physician medical title.

^h^ETitle: physician education title.

^i^POR: physician online reputation.

^j^OMR: offline medical record.

^k^N/A: not applicable.

#### Results for Information Continuity

Table 2 results suggest that offline experience negatively affects physician response speed (*β*=–.009, *P*=.053) and information quality (*β*=–.518, *P*<.001). Offline experience positively influences interaction quality (*β*=.006, *P*=.02). For offline experience provision, hypotheses 1a and 1c are supported but hypothesis 1b is not supported. Table 3 results show that offline medical record provision negatively affects physician response speed (*β*=–.001, *P*<.001), information quality (*β*=–.025, *P*<.001), and interaction quality (*β*=–.009, *P*<.001). For offline medical records provision, hypothesis 1a is supported but hypotheses 1b and 1c are not supported. The results in Table 4 present that offline detailed information provision positively affects physician response speed (*β*=.020, *P*<.001), information quality (*β*=.797, *P*<.001), and interaction quality (*β*=.042, *P*<.001). For offline detailed information provision, hypothesis 1a is not supported but hypotheses 1b and 1c are supported. Thus, hypotheses 1a, 1b, and 1c are partly supported.

#### Results for Interpersonal Continuity

The influences of interpersonal continuity on physician service are shown in Table S2 of Multimedia Appendix 2. We find that interpersonal continuity negatively moderates the relationship between offline experience provision and response speed (*β*=–.034, *P*=.006) and the relationship between offline experience provision and information quality (*β*=–.555, *P*<.001). We also find that interpersonal continuity positively moderates the relationship between offline medical record provision and interaction quality (*β*=–.010, *P*<.001), the relationship between offline detailed information provision and information quality (*β*=.015, *P*<.001), and the relationship between offline detailed information provision and interaction quality (*β*=.016, *P*=.01). Thus, for interpersonal continuity, hypothesis 2 is partly supported.

### Empirical Results: Long-term Effects

The Probit regression was used to obtain our long-term effect results, which are shown in Tables 5 and 6. The results for information continuity are shown in Table 5. Our results suggest that offline experience positively affects physician response speed (*β*=.006, *P*=.04), information quality (*β*=.001, *P*<.001), and interaction quality (*β*=.602, *P*<.001). For the long-term effects of information continuity, hypothesis 1d is supported. The results for interpersonal continuity are shown in Table 6. The results indicate that the interpersonal continuity only positively moderates the relationship between offline detailed information and repeat purchase (*β*=.143, *P*=.04). Thus, hypothesis 2 is partly supported.

**Table 5:** Results for information continuity: long-term effects.

Variables	Offline experience					Offline medical record						Offline detailed information				
	Model 1^a^		Model 2^b^			Model 1^c^			Model 2^d^			Model 1^e^			Model 2^f^	
	*β* (SD)	*P* value	*β* (SD)	*P* value	*β* (SD)		*P* value	*β* (SD)		*P* value	*β* (SD)		*P* value	*β* (SD)		*P* value
Level	.014 (.003)	.001	.014 (.003)	.001	.014 (.004)		.001	.012 (.004)		.004	.351 (.157)		.03	.402 (.153)		<.001
MTitle1^g^	.001 (.004)	.78	<.001 (.004)	.95	.001 (.005)		.78	<.001 (.005)		.97	–1.701 (.185)		<.001	–1.732 (.179)		<.001
MTitle2	.003 (.004)	.49	.003 (.004)	.52	.003 (.005)		.49	.003 (.005)		.52	–.243 (.183)		.18	–.369 (.177)		.04
ETitle^h^	.007 (.003)	.14	.007 (.003)	.14	.007 (.004)		.14	.006 (.004)		.20	–.017 (.162)		.92	.101 (.158)		.52
POR^i^	–.068 (.005)	<.001	–.068 (.005)	<.001	–.068 (.007)		<.001	–.057 (.008)		<.001	–.418 (.257)		.10	–1.988 (.261)		<.001
OE^j^	N/A^k^	N/A	.006 (.002)	.04	N/A		N/A	N/A		N/A	N/A		N/A	N/A		N/A
OMR^l^	N/A	N/A	N/A	N/A	N/A		N/A	.001 (.000)		<.001	N/A		N/A	N/A		N/A
ODI^m^	N/A	N/A	N/A	N/A	N/A		N/A	N/A		N/A	N/A		N/A	.602 (.028)		<.001

^a^Adjusted *R*^2^=0.013; *F*_5,7720_=21.064; *P*<.001.

^b^Adjusted *R*^2^=0.013; *F*_1,7719_=4.162; *P*=.04.

^c^Adjusted *R*^2^=0.013; *F*_5,7720_=21.064; *P*<.001.

^d^Adjusted *R*^2^=0.014; *F*_1,7719_=12.792; *P*<.001.

^e^Adjusted *R*^2^=0.025; *F*_5,7720_=40.078; *P*<.001.

^f^Adjusted *R*^2^=0.079; *F*_1,7719_=455.791; *P*<.001.

^g^MTitle1: physician medical title.

^h^ETitle: physician education title.

^i^POR: physician online reputation.

^j^OE: offline experience.

^k^N/A: not applicable.

^l^OMR: offline medical record.

^m^ODI: offline detailed information.

**Table 6:** Results for interpersonal continuity: long-term effects.

Variables	Offline experience^a^			Offline medical records^b^			Offline detailed information^c^	
	*β* (SD)	*P* value	*β* (SD)		*P* value	*β* (SD)		*P* value
Level	.012 (.005)	.02	.011 (.005)		.03	.467 (.156)		.003
MTitle1^d^	.000 (.005)	.97	.000 (.005)		.98	–1.738 (.179)		<.001
MTitle2	.003 (.005)	.54	.003 (.005)		.52	–.372 (.177)		.04
ETitle^e^	.007 (.004)	.14	.006 (.004)		.20	.108 (.158)		.49
POR^f^	–.069 (.007)	<.001	–.057 (.008)		<.001	–1.960 (.261)		<.001
OE^g^	–.001 (.008)	.86	N/A^h^		N/A	N/A		N/A
SP^i^×OE	.009 (.008)	.28	N/A		N/A	N/A		N/A
OMR^j^	N/A	N/A	.000 (.000)		.05	N/A		N/A
SP×OMR	N/A	N/A	6.457E-5 (.000)		.65	N/A		N/A
ODI^k^	N/A	N/A	N/A		N/A	.523 (.066)		<.001
SP×ODI	N/A	N/A	N/A		N/A	.143 (.070)		.04

^a^Adjusted *R*^2^=0.013.

^b^Adjusted *R*^2^=0.014.

^c^Adjusted *R*^2^=0.079.

^d^MTitle1: physician medical title.

^e^ETitle: physician education title.

^f^POR: physician online reputation.

^g^OE: offline experience.

^h^N/A: not applicable.

^i^SP: same physician.

^j^OMR: offline medical record.

^k^ODI: offline detailed information.

### Robustness Check

In the main analysis, we did not consider whether a physician has also provided team service. As we only focused on individual service, we only included those physicians who did not provide team service; 25 physicians who provided team service were deleted. We used the new data to obtain empirical results (given the limited space, the robustness check results are included), and consistent results were found. Our results appear to be robust.

## Discussion

### Overview

Based on the information richness theory and continuity of care, this study investigates both short-term and long-term effects of information continuity and interpersonal continuity on physician service online by collecting data of 7200 patients with 360 physicians covering complete interaction records from a professional online platform in China. Our findings have theoretical and practical support for web-based managers and service providers to improve medical service quality.

### Results Analysis

By collecting a data set from Haodf.com, we confirm the effects of information continuity and interpersonal continuity on the changing physician service. The summary of the results is shown in Table S3 and Table S4 of Multimedia Appendix 2. Our empirical study generated several important results.

First, both short-term and long-term effects of information continuity and interpersonal continuity were found. Continuity of care is important for medical service [1]. There is little understanding of how to improve the continuity of care and the effects of continuity of care. We find that providing offline experience is useful for improving the continuity of care and is helpful for physicians for providing high-quality service.

Second, the effects of information continuity showed heterogeneity. Offline experience and medical record provision are helpful for a physician to improve the response speed. However, detailed information provision increases response time. Offline experience and medical records could help refresh a physician’s memory of the patient and then reply quickly. However, detailed offline information is written and provided by patients; therefore, it may contain a patient’s personalized feelings, experience, and other questions, which takes the physician time to understand and then give a detailed reply to the patient’s need. The above reasons can be used to explain the effects of the 3 independent variables on information quality. For the interaction quality, offline experience and detailed information provision help improve the interaction frequency between physicians and patients; however, offline medical records provision negatively affects interaction frequency. The reasons are that (1) web-based medical records are also a type of interaction and influence the calculation of interaction and (2) medical records contain much information about a disease condition, which a physician often needs to judge the disease. Without these medical records, the physician has to interact with patients to obtain relevant information.

Third, the effects of offline experience, medical records, and detailed information provisions on repeat purchases are consistent. Information continuity is helpful for a physician’s service in the future.

Fourth, the moderating effects of interpersonal continuity were also consistent. Most of the moderating effects were positive and consistent with our hypotheses, that is, high interpersonal continuity would enhance the relationships between information continuity and physician service.

### Implications

Our study produces several insights, which have implications for continuity of care, cross-channel behavior, and online health community literature. More importantly, these insights as a whole contribute to the design of integrated medical services. For the practical implications, first, for those who design and manage online health communities, attention needs to be paid not only to facilitating the transaction but also to interaction quality. From the continuity of care perspective, we have found significant influences of offline experience provision on physician online service. Our results suggest that mechanisms that can guide patients to provide offline experience should be established. In particular, the offline detailed information provision should be emphasized. Moreover, based on the positive effects of interpersonal continuity, online health community platforms should encourage patients to choose online physicians according to their offline physicians to improve consistency and then improve interpersonal continuity. Second, for the physicians, not only the short-term effects of offline experience provision should be valued but also long-term effects have to be highly regarded. Physicians can guide patients to remember the offline experience and provide their offline information, which is helpful for the physician to provide high-quality service and increase the repurchase rate further. Third, for the patient, our results suggest that patients could go to the nearby hospital to obtain medical records or other material and then provide them to the online physician to receive a better service.

Our study contributes to the current knowledge in several ways. First, our work extends our knowledge of the effects of information technology artifacts on the health care field from the continuity of care perspective. Although relevant departments believe that the use of information technology could realize the mutual recognition of inspection results, sharing of medical records, and thus improving the continuity of care [3], there are no empirical studies to examine the true effects. Our study has investigated the role of online health community use in improving the continuity of care. Moreover, we investigated the specific measures the patients and physicians should take to improve the continuity of care.

Second, our study enriches the literature on the continuity of care. Information continuity, interpersonal continuity, and time continuity have been widely discussed in previous studies [28,29]. However, they failed to examine the effects of different continuity dimensions on physician service, especially in a web-based environment. Our results show that the different dimensions of continuity of care have different effects on physician service behavior. Moreover, there are interaction effects between information continuity and interpersonal continuity.

Third, our study provides evidence on the cross-channel context. Although many studies have examined the channel effects in health care [6,46], they mainly focus on behaviors switching from online to offline. This study focuses on the effects of offline experience on online behavior, that is, behaviors switching from offline to online. Our results show that a patient’s offline experience provision has a positive influence on the physician’s web-based service.

### Limitations of This Study

Several limitations and prospects in this study must be considered. First, we studied only 1 context, which helps us improve the internal validity, but it may also reduce the generalizability of our findings. Future studies could validate our results in other contexts. Second, word count and interaction count are used for measuring physician service. Future studies could use more accurate methods to measure physician services, such as text mining and sentiment analysis. Third, the unit of analysis is the individual online health community patient-physician interaction, and we do not have individual characteristics about patients. Future studies could try to obtain patient information and control them. Fourth, characteristics of physicians that may influence the use of web-based services are age, experience with computers/technology, and preferences toward in-person versus web-based delivery of services. Future studies could try to obtain more physician information and control them. Fifth, we assume that in-person experience and skills of physicians are transferrable to the online context. Future studies could obtain this skill of different physicians and control it.

### Conclusions

Although abundant studies have investigated online health community behaviors and cross-channel behaviors, this study is among the first to investigate the effects of information providing from the continuity of care perspective and the influence of offline experience on online behaviors. Our study offers a better understanding of online behaviors, enriches the knowledge of the effects of information technology artifacts in the health care field, and contributes to the continuity of care literature. We have reported both short-term and long-term effects of the offline medical service experience on the online medical service experience. We believe that this paper could provoke some new thoughts on online health communities.

## Appendix

Multimedia Appendix 1 Descriptive statistics and correlations of variables.

Multimedia Appendix 2 Influences of interpersonal continuity on physician service and summary data of this study.
