# Supplementary material for: The Effects of Information Continuity and Interpersonal Continuity on Physician Services Online: Cross-sectional Study
Source: JMIR Med Inform. 2022 Jul 21;10(7):e35830. doi: 10.2196/35830 (PMC9353683; doi:10.2196/35830)
Supplement: Multimedia Appendix 1 [file medinform_v10i7e35830_app1.docx]

Table S1. Descriptive statistics and correlations

|  | Mean (SD) | 1 | 2 | 3 | 4 | 5 | 6 | 7 | 8 | 9 | 10 | 11 | 12 |
| --- | --- | --- | --- | --- | --- | --- | --- | --- | --- | --- | --- | --- | --- |
| 1. *MTitle1* | N/A |  |  |  |  |  |  |  |  |  |  |  |  |
| 2. *MTitle2* | N/A | –0.738 (0.000) |  |  |  |  |  |  |  |  |  |  |  |
| 3. *ETitle* | N/A | 0.488 (0.000) | –0.362 (0.000) |  |  |  |  |  |  |  |  |  |  |
| 4. *Level* | N/A | 0.104 (0.000) | –0.085 (0.000) | 0.162 (0.000) |  |  |  |  |  |  |  |  |  |
| 5. *POR* | 3.615 (0.272) | 0.320 (0.000) | –0.168 (0.000) | 0.418 (0.000) | 0.434 (0.000) |  |  |  |  |  |  |  |  |
| 6. *OE* | N/A | 0.155 (0.000) | –0.099 (0.000) | 0.082 (0.000) | –0.001 (0.940) | 0.061 (0.000) |  |  |  |  |  |  |  |
| 7. *OMR* | N/A | –0.027 (0.016) | –0.007 (0.551) | –0.082 (0.000) | –0.111 (0.000) | –0.396 (0.000) | 0.289 (0.000) |  |  |  |  |  |  |
| 8. *ODI* | 38.50 (94.687) | 0.065 (0.000) | –0.003 (0.786) | 0.089 (0.000) | 0.123 (0.000) | 0.314 (0.000) | –0.141 (0.000) | –0.357 (0.000) |  |  |  |  |  |
| 9. *SP* | N/A | 0.137 (0.000) | –0.093 (0.000) | 0.183 (0.000) | 0.686 (0.000) | 0.418 (0.000) | 0.250 (0.000) | –0.049 (0.000) | 0.117 (0.000) |  |  |  |  |
| 10. *RS* | 0.673 (0.373) | –0.051 (0.013) | 0.049 (0.018) | –0.021 (0.303) | 0.018 (0.380) | 0.138 (0.000) | –0.049 (0.017) | –0.227 (0.000) | 0.317 (0.000) | –0.005 (0.820) |  |  |  |
| 11. *InfQ* | 12.609 (27.48)7 | 0.068 (0.000) | –0.007 (0.531) | 0.091 (0.000) | 0.121 (0.000) | 0.296 (0.000) | –0.136 (0.000) | –0.338 (0.000) | 0.985 (0.000) | 0.115 (0.000) | 0.288 (0.000) |  |  |
| 12. *IntQ* | 17.97 (491.045) | –0.039 (0.000) | 0.025 (0.026) | –0.037 (0.000) | –0.019 (0.087) | –0.135 (0.000) | 0.020 (0.080) | 0.104 (0.000) | –0.088 (0.000) | –0.021 (0.065) | –0.056 (0.000) | –0.077 (0.000) |  |
| 13. *RP* | 0.29 (0.030) | –0.032 (0.000) | 0.020 (0.085) | –0.031 (0.000) | –0.015 (0.174) | –0.109 (0.000) | 0.016 (0.164) | 0.084 (0.000) | –0.093 (0.000) | –0.018 (0.107) | –0.059 (0.000) | –0.082 (0.000) | 0.978 (0.000) |

Notes: Physician medical titles (MTitle1 and MTitle2), Physician education title (ETitle), Physician online reputation (POR), Hospital level (Level), Offline experience (OE), Offline medical records (OMR), Offline detailed information (ODI), Same physician (SP), Response speed (RS), Information quality (InfQ), Interaction quality (IntQ), Repeat purchase (RP).
