# Supplementary material for: The Effects of Information Continuity and Interpersonal Continuity on Physician Services Online: Cross-sectional Study
Source: JMIR Med Inform. 2022 Jul 21;10(7):e35830. doi: 10.2196/35830 (PMC9353683; doi:10.2196/35830)
Supplement: Multimedia Appendix 2 [file medinform_v10i7e35830_app2.docx]

**Table S2.** Results for interpersonal continuity: short-term effects

| Variables | Response speed | | | | | | Information quality | | | | | | Interaction quality | | | | | |
| --- | --- | --- | --- | --- | --- | --- | --- | --- | --- | --- | --- | --- | --- | --- | --- | --- | --- | --- |
|  | Mode 1 | | Model 2 | | Model 3 | | Mode 1 | | Model 2 | | Model 3 | | Mode 1 | | Model 2 | | Model 3 | |
|  | *β* (SD) | *P* | *β* (SD) | *P* | *β* (SD) | *P* | *β* (SD) | *P* | *β* (SD) | *P* | *β* (SD) | *P* | *β* (SD) | *P* | *β* (SD) | *P* | *β* (SD) | *P* |
| Level | –.009  (0.007) | 0.228 | –.008  (0.008) | 0.280 | –.013  (0.006) | 0.037 | 0.090  (0.055) | 0.102 | 0.046  (0.057) | 0.425 | 0.039  (0.009) | 0.000 | 0.020  (0.004) | 0.000 | 0.290  (0.057) | 0.000 | 0.075  (0.015) | 0.000 |
| MTitle1 | –.019  (0.008) | 0.014 | –.017  (0.008) | 0.029 | –.022  (0.007) | 0.003 | 0.159  (0.058) | 0.006 | 0.150  (0.057) | 0.008 | 0.023  (0.011) | 0.034 | 0.010  (0.004) | 0.009 | –.090  (0.057) | 0.000 | –.138  (0.017) | 0.000 |
| MTitle2 | –.001  (0.007) | 0.876 | –.001  (0.007) | 0.877 | –.006  (0.007) | 0.397 | 0.195  (0.057) | 0.001 | 0.185  (0.056) | 0.001 | 0.003  (0.011) | 0.763 | 0.009  (0.004) | 0.025 | –.006  (0.056) | 0.712 | –.025  (0.017) | 0.134 |
| ETitle | –.017  (0.007) | 0.012 | –.014  (0.007) | 0.038 | –.013  (0.007) | 0.054 | –.119  (0.051) | 0.020 | –.054  (0.050) | 0.285 | 0.037  (0.009) | 0.000 | 0.002  (0.003) | 0.527 | 0.029  (0.050) | 0.039 | 0.012  (0.015) | 0.426 |
| POR | 0.093  (0.011) | 0.000 | 0.059  (0.011 | 0.000 | 0.040  (0.011) | 0.000 | 1.962  (0.081) | 0.000 | 1.146  (0.087) | 0.000 | –.146  (0.016) | 0.000 | –.105  (0.005) | 0.000 | –.450  (0.087) | 0.000 | –.316  (0.024) | 0.000 |
| OE | 0.021  (0.012) | 0.080 |  |  |  |  | –.028  (0.091) | 0.761 |  |  |  |  | –.001  (0.006) | 0.851 |  |  |  |  |
| SP × OE | –0.034  (0.012) | 0.006 |  |  |  |  | –.555  (0.094) | 0.000 |  |  |  |  | 0.008  (0.006) | 0.204 |  |  |  |  |
| OMR |  |  | –.001  (0.000) | 0.000 |  |  |  |  | –.025  (0.002) | 0.000 |  |  |  |  | –.001  (0.002) | 0.121 |  |  |
| SP × OMR |  |  | 0.000  (0.000) | 0.215 |  |  |  |  | 0.000  (0.002) | 0.824 |  |  |  |  | –.010  (0.002) | 0.000 |  |  |
| ODI |  |  |  |  | 0.023  (0.003) | 0.000 |  |  |  |  | 0.710  (0.004) | 0.000 |  |  |  |  | 0.036  (0.006) | 0.000 |
| SP × ODI |  |  |  |  | –.004  (0.003) | 0.163 |  |  |  |  | 0.015  (0.004) | 0.000 |  |  |  |  | 0.016  (0.007) | 0.014 |
| Adjusted R^2^ | 0.010 | | 0.017 | | 0.047 | | 0.117 | | 0.146 | | 0.370 | | 0.052 | | 0.192 | | 0.068 | |

Notes: Robust S.E. are in parentheses.

**Table S3.** Results summary.

| Variables | Response speed | Information quality | Interaction quality | Repeat purchase |
| --- | --- | --- | --- | --- |
| Offline experience | Negative | Negative | Negative | Negative |
| Offline medical record | Negative | Negative | Negative | Positive |
| Offline detailed information | Positive | Positive | Positive | Positive |
| Same physician × offline experience | Negative | Negative | No effect | No effect |
| Same physician × offline medical record | No effect | No effect | Negative | No effect |
| Same physician × offline detailed information | No effect | Positive | Positive | Positive |

**Table S4.** Hypotheses summary.

| Hypotheses | Content | Support or not |
| --- | --- | --- |
| H1a | (Short-term) high information continuity would improve the response speed of a physician’s reply. | Partly support |
| H1b | (Short-term) high information continuity would improve the information quality of a physician’s reply. | Partly support |
| H1c | (Short-term) high information continuity would improve the interaction quality of a physician’s reply. | Partly support |
| H1d | (Long-term) high information continuity would increase a patient’s repeat purchase. | Support |
| H2 | High interpersonal continuity would enhance the relationships between information continuity and a physician’s service. | Partly support |
